# CircRNA DOCK1 Regulates miR-409-3p/MCL1 Axis to Modulate Proliferation and Apoptosis of Human Brain Vascular Smooth Muscle Cells

**DOI:** 10.3389/fcell.2021.655628

**Published:** 2021-05-24

**Authors:** Xinmin Ding, Xiaolong Wang, Li Han, Zhiyu Zhao, Shuai Jia, Yuanzhao Tuo

**Affiliations:** Department of Neurosurgery, Shanxi Bethune Hospital, The Third Hospital of Shanxi Medical University, Taiyuan, China

**Keywords:** intracranial aneurysm, brain vascular smooth muscle cell, circ_DOCK1, miR-409-3p, MCL1, H_2_O_2_

## Abstract

**Background:**

Intracranial aneurysm is an abnormal expansion in the intracranial arteries, which is associated with growth and apoptosis of vascular smooth muscle cells. Circular RNAs (circRNAs) have implicated in the progression of intracranial aneurysms. The purpose of this paper is to study the function and mechanism of circRNA dedicator of cytokinesis 1 (circ_DOCK1) in regulating proliferation and apoptosis of human brain vascular smooth muscle cells (HBVSMCs).

**Methods:**

HBVSMCs were exposed to hydrogen peroxide (H_2_O_2_). Cell proliferation and apoptosis were detected by 3-(4,5-dimethylthiazol-2-yl)-2,5-diphenyl tetrazolium bromide (MTT) and flow cytometry, respectively. Circ_DOCK1, microRNA (miR)-409-3p, and myeloid cell leukemia sequence 1 (MCL1) levels were examined by quantitative reverse transcription polymerase chain reaction or western blotting. The target association was assessed by dual-luciferase reporter, RNA pull-down, and RNA immunoprecipitation assays.

**Results:**

Exposure to H_2_O_2_ decreased proliferation and increased apoptosis of HBVSMCs. Circ_DOCK1 expression was reduced in H_2_O_2_-treated HBVSMCs. Circ_DOCK1 overexpression rescued H_2_O_2_-caused reduction of proliferation and PCNA expression and attenuated H_2_O_2_-induced apoptosis and expression of Bcl-2, Bax, and cleaved PARP. MiR-409-3p was targeted by circ_DOCK1 and upregulated in H_2_O_2_-treated HBVSMCs. MiR-409-3p upregulation mitigated the role of circ_DOCK1 in proliferation and apoptosis of H_2_O_2_-treated HBVSMCs. MCL1 was targeted *via* miR-409-3p and downregulated *via* H_2_O_2_ treatment. Circ_DOCK1 overexpression enhanced MCL1 expression *via* modulating miR-409-3p. MiR-409-3p knockdown weakened H_2_O_2_-induced proliferation reduction and apoptosis promotion *via* regulating MCL1.

**Conclusion:**

Circ_DOCK1 overexpression mitigated H_2_O_2_-caused proliferation inhibition and apoptosis promotion in HBVSMCs by modulating miR-409-3p/MCL1 axis.

## Introduction

Intracranial aneurysm is an abnormal expansion in the intracranial arteries which could lead to aneurysm rupture (Brinjikji et al., 2016). The therapeutic strategies against intracranial aneurysm mainly include surgical and endovascular approaches (Lozano et al., 2019). However, the majority of cases with vascular remodeling undergo eventual rupture (Frosen et al., 2004). Smooth muscle cells are responsible in maintaining the vascular structure and are associated with cerebrovascular diseases, including intracranial aneurysm (Frosen and Joutel, 2018). The vascular smooth muscle cell apoptosis can lead to the degradation of vascular wall, thus inducing the development and rupture of intracranial aneurysm (Liu Z. et al., 2019). Hence, exploring the mechanism of vascular smooth muscle cell proliferation and apoptosis may help in finding novel ways for intracranial aneurysm treatment.

Non-coding RNAs are important regulators for vascular smooth muscle cell processes in vascular diseases (Leeper and Maegdefessel, 2018). Circular RNAs (circRNAs) are a type of non-coding RNAs without 5′ and 3′ ends, which can function as microRNA (miRNA) sponges to take part in the regulation of vascular smooth muscle cell processes in intracranial aneurysm (Huang et al., 2019). For instance, hsa_circ_0021001 can act as a potential biomarker for intracranial aneurysm, and patients with low expression of hsa_circ_0021001 have the worse outcomes (Teng et al., 2017). The circRNA dedicator of cytokinesis 1 (circ_DOCK1, also called hsa_circ_0020397 according to the circRNA ID of circBase database) is downregulated in artery wall tissues and vascular smooth muscle cells of intracranial aneurysm patients, and it promotes vascular smooth muscle cell proliferation (Wang et al., 2019; Yin and Liu, 2021). Although the reports also uncovered the miR-138/KDR and miR-502-5p/GREM1 networks underlying the regulation of circ_DOCK1, our understanding of its molecular basis is still limited.

MiRNAs are a group of short non-coding RNAs that modulate mRNA expression, which are involved in intracranial aneurysm progression (Liu et al., 2014) and are associated with the regulation of vascular smooth muscle cell proliferation and apoptosis (Wang and Atanasov, 2019). For instance, miR-448-3p and miR-205 are associated with the progression of intracranial aneurysm (Zhang et al., 2018; Zhong et al., 2019). Furthermore, miR-409-3p is a differentially expressed miRNA in intracranial aneurysm (Bekelis et al., 2016). Nevertheless, the function and mechanism of miR-409-3p in vascular smooth muscle cell dysfunction in intracranial aneurysm remains unknown.

Myeloid cell leukemia sequence 1 (MCL1) is a key member of B cell lymphoma-2 (Bcl-2) prosurvival family, which controls cell proliferation and apoptosis (Ertel et al., 2013). Furthermore, MCL1 contributes to vascular smooth muscle cell proliferation and inhibits apoptosis in vascular diseases, including intracranial aneurysm (Lee et al., 2015; Zhao W. et al., 2018). CircInteractome and starBase algorithms predict miR-409-3p might bind to circ_DOCK1 and MCL1. Thus, we hypothesized circ_DOCK1 might indirectly regulate MCL1 by miR-409-3p to participate in the regulation of vascular smooth muscle cell dysfunction in intracranial aneurysm.

Oxidative stress is well known as a contributor to the development and rupture of intracranial aneurysm (Starke et al., 2013). Hydrogen peroxide (H_2_O_2_), an inducer of oxidative stress, is involved in apoptosis of vascular smooth muscle cells (Meng et al., 2018). Moreover, H_2_O_2_ has been used to establish an *in vitro* of intracranial aneurysm *via* inducing the apoptosis of vascular smooth muscle cells (Zhao W. et al., 2018; Shi et al., 2019). In this study, we established the cellular model of intracranial aneurysm using H_2_O_2_-treated human brain vascular smooth muscle cells (HBVSMCs). Moreover, we analyzed the function of circ_DOCK1 on H_2_O_2_-caused HBVSMC dysfunction and explored the potential regulatory network of circ_DOCK1/miR-409-3p/MCL1. This study may propose novel insight into the vascular smooth muscle cell dysfunction in intracranial aneurysm.

## Materials and Methods

### Cell Culture and H_2_O_2_ Treatment

Human brain vascular smooth muscle cells (Cat. No. CP-H116) were purchased from Procell (Wuhan, China) and cultured in specific complete medium for vascular smooth muscle cell culture (Cat. No. CM-H116; Procell) at 37°C and 5% CO_2_. To establish an *in vitro* of intracranial aneurysm as reported (Zhao W. et al., 2018; Shi et al., 2019), cells were incubated with 0, 30, 90, or 180 μM of H_2_O_2_ (Sigma, St. Louis, MO, United States) for 6 h.

### Cell Transfection

Circular RNAs dedicator of cytokinesis 1 overexpression vector was constructed by Geneseed (Guangzhou, China), and the pCD5-ciR vector was regarded as a negative control (vector). MiR-409-3p mimic, mimic negative control (miR-NC), miR-409-3p inhibitor (anti-miR-409-3p), inhibitor negative control (anti-miR-NC), small interfering RNA (siRNA) for MCL1 (si-MCL1), and negative control of siRNA (si-NC) were generated by Genomeditech (Shanghai, China), and the oligonucleotide sequences are shown in Table 1. For cell transfection, HBVSMCs were incubated with 1 μg constructed vectors or 30 nM oligonucleotides and 5 μl Lipofectamine 2000 (Thermo Fisher Scientific, Waltham, MA, United States). After 24 h, transfected cells were harvested for expression analysis or subjected to H_2_O_2_ (180 μM) exposure.

**TABLE 1:** The sequences of oligonucleotides used in this study.

Name	Sequence (5′-3′)
si-MCL1	AAAAGCUUCCCUUGUACAGUA
si-NC	AAGACAUUGUGUGUCCGCCTT
miR-409-3p mimic	GAAUGUUGCUCGGUGAACCCCU
miR-NC	CGAUCGCAUCAGCAUCGAUUGC
Anti-miR-409-3p	AGGGGUUCACCGAGCAACAUUC
Anti-miR-NC	CUAACGCAUGCACAGUCGUACG

### Quantitative Reverse Transcription Polymerase Chain Reaction

Human brain vascular smooth muscle cells were lysed in Trizol (Thermo Fisher Scientific), and total RNA was isolated following the accompanying instructions. Then 1 μg RNA was reverse transcribed using miRNA Reverse Transcriptase kit or M-MLV Reverse Transcriptase kit (Thermo Fisher Scientific) according to the accompanying instructions. For quantitative reverse transcription polymerase chain reaction (qRT-PCR) analysis, cDNA was mixed with SYBR (Vazyme, Nanjing, China) and designed primers. The primer pairs were synthesized by Sangon (Shanghai, China), and the sequences are shown in Table 2. The qRT-PCR was performed on CFX96^TM^ Real-time PCR Detection System (Bio-Rad Laboratories, Hercules, CA, United States). Relative expression level was detected by the 2^–ΔΔ*Ct*^ method with U6 or glyceraldehyde 3-phosphate dehydrogenase (GAPDH) as an internal control.

**TABLE 2:** The sequences for primers used for qRT-PCR.

Name	Sequence (5′-3′)	
	Forward	Reverse
miR-409-3p	GCCGAGGAATGTTGCTCGGTG	CTCAACTGGTGTCGTGGA
U6	CTCGCTTCGGCAGCACA	AACGCTTCACGAATTTGCGT
circ_DOCK1	GTGAACCGAACCGTCATTTC	CCTCGGTACCACCCTTCATA
DOCK1	ATGAAGCCTCATCCCCTCTTT	TCACCCGGGATGACTGTTTC
MCL1	GCCTTCCAAGGATGGGTTTG	AGGTTGCTAGGGTGCAACTC
GAPDH	TTCTTTTGCGTCGCCAGGTG	GGAGGGAGAGAACAGTGAGC

### RNase R Digestion and Actinomycin D Analyses

The circular structure of circ_DOCK1 was analyzed by RNase R digestion and actinomycin D analyses. For RNase R digestion analysis, RNA was treated with 2 U/μg RNase R (Geneseed) for 20 min, followed by reverse transcription and qRT-PCR for detection of circ_DOCK1 and linear DOCK1 expression.

For actinomycin D analysis, HBVSMCs were challenged by 2 μg/ml actinomycin D (Sigma) for 0, 8, 16, or 24 h, followed by collection for RNA isolation. The isolated RNA was used for qRT-PCR to measure circ_DOCK1 and linear DOCK1 expression.

### 3-(4,5-Dimethylthiazol-2-yl)-2,5-Diphenyl Tetrazolium Bromide

Cell proliferation was analyzed by 3-(4,5-dimethylthiazol-2-yl)-2,5-diphenyl tetrazolium bromide (MTT). After the treatment of H_2_O_2_ or not, 1 × 10^4^ HBVSMCs were placed in 96-well plates. After incubation for 0, 24, or 48 h, 10 μl 5 mg/ml MTT (Solarbio, Beijing, China) was added, and cells were continuously cultured for 4 h. The medium was then discarded, and 100 μl dimethyl sulfoxide (DMSO) (Beyotime, Shanghai, China) was added. Optical density (OD) value at 570 nm was determined *via* a microplate reader (Bio-Rad Laboratories).

### Flow Cytometry

Cell apoptosis was measured with an Annexin V-fluorescein isothiocyanate (FITC) apoptosis detection kit (Beyotime) following the instruction. After exposure to H_2_O_2_ or not, 2 × 10^5^ HBVSMCs were added in 12-well plates and cultured for 48 h. Next, cells were collected, interacted with Annexin V-binding buffer, and then dyed with 10 μl Annexin V-FITC and propidium iodide (PI). The apoptotic cells (with Annexin V-FITC positive and PI positive or negative) were measured with a flow cytometer (Agilent, Beijing, China).

### Western Blotting

Human brain vascular smooth muscle cells were lysed in RIPA buffer (Beyotime), and protein was obtained after a centrifugation at 10,000 × *g* for 5 min. The protein was quantified with a bicinchoninic acid kit (Thermo Fisher Scientific) according to the instructions. The samples (20 μg) were separated by a sodium dodecyl sulfate-polyacrylamide gel electrophoresis, and then transferred on nitrocellulose membrane (Bio-Rad Laboratories). The membranes were incubated in 3% bovine serum albumin (Solarbio) for 1 h, and then interacted with primary antibodies overnight and secondary antibody for 2 h. All antibodies were purchased from Abcam (Cambridge, United Kingdom), including proliferating cell nuclear antigen (PCNA) (ab152112, 1:2,000 dilution), Bcl-2 (ab194583, 1:500 dilution), Bcl-2-associated X (Bax) (ab53154, 1:500 dilution), cleaved poly-ADP ribose polymerase (PARP) (ab32064, 1:3,000 dilution), MCL1 (ab243136, 1:2,000 dilution), GAPDH (ab9485, 1:5,000 dilution), and horseradish peroxidase-labeled IgG (ab6721, 1:10,000 dilution). Next, the membranes were interacted with enhanced chemiluminescence (Solarbio), and the blots were analyzed *via* Quantity One software (Bio-Rad Laboratories) with GAPDH as a normalized reference.

### Dual-Luciferase Reporter Assay

The binding sites of miRNAs to circ_DOCK1 were predicted by the web-based program CircInteractome^1^. The molecular targets of miR-409-3p were predicted using the online database starBase (which are based on miRNA target prediction programs, i.e., TargetScan, miRanda, microT, PITA, miRmap, and PicTar)^2^. The wild-type (WT) sequence (…AACAUU…) of circ_DOCK1 or MCL1 was inserted in the pmir-GLO vector (Promega, Madison, WI, United States), generating the circ_DOCK1-WT and MCL1-WT luciferase reporter vectors. The mutant (MUT) luciferase reporter vectors (circ_DOCK1-MUT and MCL1-MUT) were constructed using the mutated sequence (…CCACGG…). These luciferase reporter vectors and miR-409-3p mimic or miR-NC were cotransfected into HBVSMCs. After 24 h, luciferase activity was measured with a dual-luciferase analysis kit (Promega).

### RNA Pull-Down and RNA Immunoprecipitation Assays

A Pierce^TM^ Magnetic RNA-Protein Pull-Down kit (Thermo Fisher Scientific) was used for RNA pull-down assay. Briefly, the biotin-labeled circ_DOCK1-WT, circ_DOCK1-MUT, and negative control (bio-NC) were generated and interacted with the magnetic beads. HBVSMCs were lysed and incubated with the magnetic beads for 8 h. MiR-409-3p level enriched on the beads was detected by qRT-PCR.

A Magna RIP^TM^ RNA-Binding Protein Immunoprecipitation kit (Sigma) was used for RNA immunoprecipitation (RIP) analysis. In brief, HBVSMC lysates were incubated with anti-Ago2 or anti-IgG-coated magnetic beads for 6 h. MCL1 and miR-409-3p levels enriched on the beads were measured *via* qRT-PCR.

### Statistical Analysis

All experiments were repeated three times with four replicates. Results were expressed as mean ± standard deviation (SD). Statistical analysis was processed by GraphPad Prism 8 (GraphPad Inc., La Jolla, CA, United States) and SPSS version 19 software (SPSS Inc., Chicago, IL, United States). The difference was compared by Student’s *t* test or one-way analysis of variance followed by Tukey’s *post hoc* test, as appropriate. It was statistically significant at *P* < 0.05.

## Results

### Circ_DOCK1 Expression Is Reduced in H_2_O_2_-Treated HBVSMCs

**FIGURE 1:**
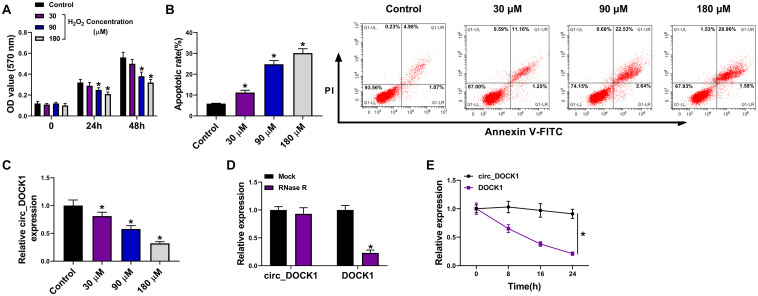
Circ_DOCK1 expression in H_2_O_2_-treated HBVSMCs. **(A)** Cell proliferation was investigated by MTT in HBVSMCs after stimulation of different doses of H_2_O_2_ or not. **(B)** Cell apoptosis was examined *via* flow cytometry in HBVSMCs after exposure to different doses of H_2_O_2_ or not. **(C)** Circ_DOCK1 abundance was detected by qRT-PCR in HBVSMCs after treatment of different doses of H_2_O_2_ or not. **(D,E)** Circ_DOCK1 and linear DOCK1 abundances were detected after incubation of RNase R or actinomycin D. *P < 0.05.

To analyze whether circ_DOCK1 was involved in intracranial aneurysms, a H_2_O_2_-caused cellular model was established using HBVSMCs. As shown in Figures 1A,B, stimulation of H_2_O_2_ led to obvious proliferation reduction and apoptosis promotion in a concentration-dependent pattern, suggesting the successful establishment of the *in vitro* model. Moreover, circ_DOCK1 expression was examined in this model. Results displayed that circ_DOCK1 abundance was evidently decreased in HBVSMCs after treatment of H_2_O_2_ in a dose-dependent pattern (Figure 1C). Additionally, the stability of circ_DOCK1 was analyzed *via* RNase R digestion and actinomycin D analyses. Circ_DOCK1, rather than DOCK1, was resistant to RNase R and actinomycin D, indicating circ_DOCK1 had a stable circular structure (Figures 1D,E). These results suggested that the downregulated circ_DOCK1 might be associated with H_2_O_2_-induced HBVSMC injury.

### Circ_DOCK1 Overexpression Attenuates H_2_O_2_-Induced HBVSMC Injury

**FIGURE 2:**
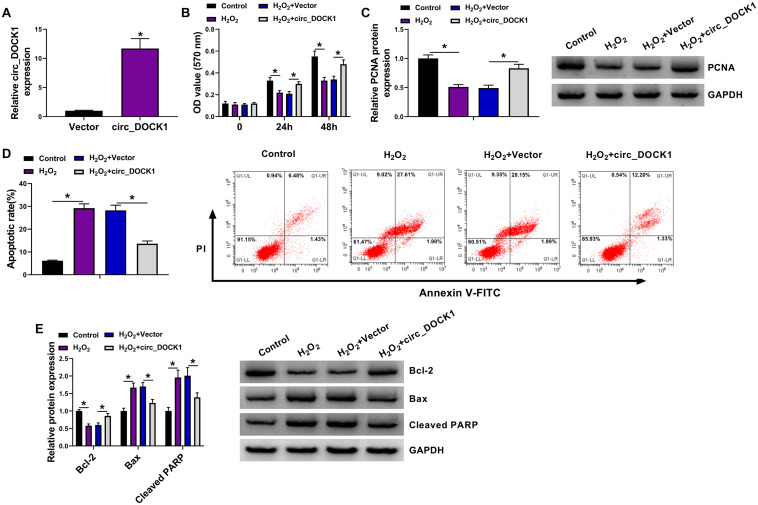
The effect of circ_DOCK1 on proliferation and apoptosis of HBVSMCs under H_2_O_2_. **(A)** Circ_DOCK1 abundance was examined in HBVSMCs transfected with vector or circ_DOCK1 overexpression vector. Cell proliferation **(B)**, PCNA expression **(C)**, apoptosis **(D)**, and levels of Bcl-2, Bax, and cleaved PARP **(E)** were determined in HBVSMCs transfected with vector or circ_DOCK1 overexpression vector before treatment of H_2_O_2_ or not. *P < 0.05.

To study the function of circ_DOCK1 in H_2_O_2_-induced model, HBVSMCs were transfected with vector or circ_DOCK1 overexpression vector before the stimulation of H_2_O_2_. The transfection of circ_DOCK1 overexpression vector markedly elevated circ_DOCK1 abundance in HBVSMCs (Figure 2A). Furthermore, circ_DOCK1 overexpression mitigated H_2_O_2_-induced decrease of cell proliferation and proliferation-related PCNA expression (Figures 2B,C). Additionally, circ_DOCK1 upregulation weakened H_2_O_2_-caused apoptosis of HBVSMCs (Figure 2D). Moreover, the antiapoptotic Bcl-2 and proapoptotic Bax and cleaved PARP levels were detected in HBVSMCs. Results showed H_2_O_2_ significantly inhibited Bcl-2 abundance and increased Bax and cleaved PARP expression, and this effect was reversed by circ_DOCK1 overexpression (Figure 2E). These results indicated circ_DOCK1 mitigated H_2_O_2_-induced HBVSMC damage.

### MiR-409-3p Is Targeted by circ_DOCK1 and Upregulated in H_2_O_2_-Treated HBVSMCs

**FIGURE 3:**
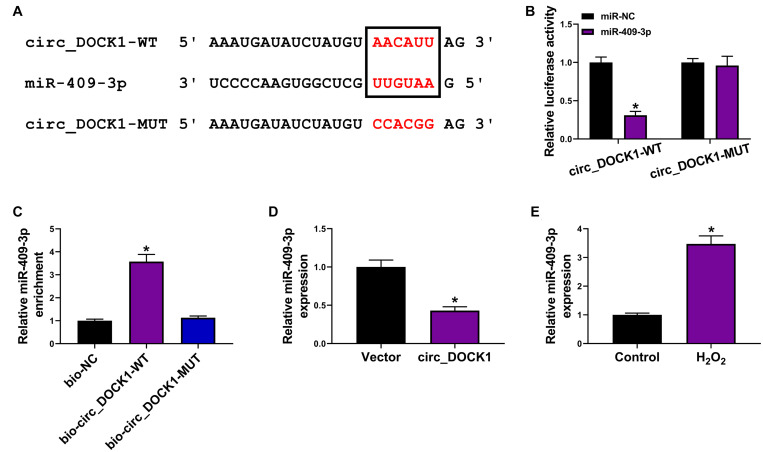
The target association of circ_DOCK1 and miR-409-3p in HBVSMCs. **(A)** The binding sequence of circ_DOCK1 and miR-409-3p was predicted by CircInteractome. **(B)** Luciferase activity of circ_DOCK1-WT and circ_DOCK1-MUT vectors was examined in HBVSMCs with transfection of miR-NC or miR-409-3p mimic. **(C)** MiR-409-3p enrichment was detected after RNA pull-down. **(D)** MiR-409-3p expression was examined in HBVSMCs transfected with vector or circ_DOCK1 overexpression vector. **(E)** MiR-409-3p abundance was examined in HBVSMCs after stimulation of H_2_O_2_ or not. *P < 0.05.

To explore the regulatory mechanism addressed by circ_DOCK1, the downstream miRNAs were predicted by CircInteractome. MiR-409-3p was a potential target, and the target sites are shown in Figure 3A. To validate their target relationship, the circ_DOCK1-WT and circ_DOCK1-MUT vectors were constructed. Moreover, miR-409-3p mimic effectively reduced the luciferase activity of circ_DOCK1-WT, but it induced little effect on the activity of circ_DOCK1-MUT when the binding sites (AACAUU) were mutated to CCACGG (Figure 3B). In addition, miR-409-3p could enrich with bio-circ_DOCK1-WT, but little enrichment was induced in bio-circ_DOCK1-MUT (Figure 3C). Additionally, miR-409-3p abundance in HBVSMCs was markedly decreased *via* circ_DOCK1 overexpression (Figure 3D). Furthermore, miR-409-3p abundance was evidently enhanced in HBVSMCs after exposure to H_2_O_2_ (Figure 3E). These results suggested that miR-409-3p was targeted *via* circ_DOCK1.

### MiR-409-3p Overexpression Mitigates the Effect of circ_DOCK1 on Cell Proliferation and Apoptosis in H_2_O_2_-Treated HBVSMCs

**FIGURE 4:**
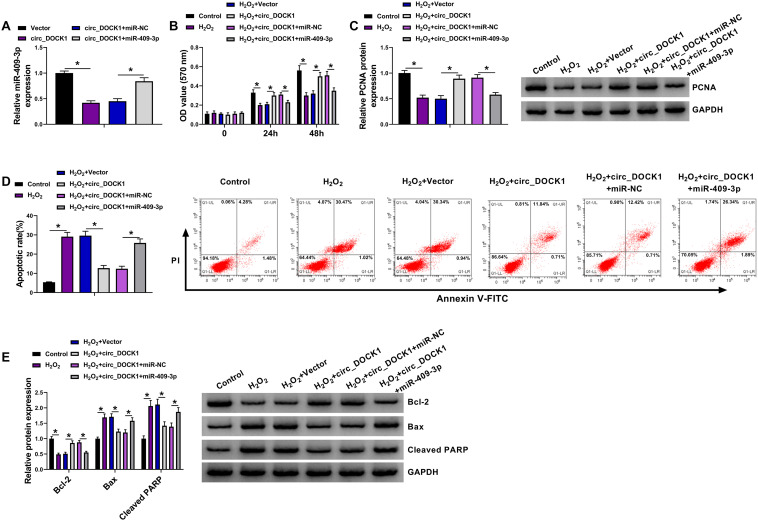
The mitigative role of miR-409-3p in circ_DOCK1-modulated regulation of proliferation and apoptosis of HBVSMCs under H_2_O_2_. **(A)** MiR-409-3p abundance was examined in HBVSMCs with transfection of vector, circ_DOCK1 overexpression vector, circ_DOCK1 overexpression vector + miR-NC, or miR-409-3p mimic. Cell proliferation **(B)**, PCNA expression **(C)**, apoptosis **(D)**, and abundances of Bcl-2, Bax, and cleaved PARP **(E)** were determined in HBVSMCs transfected with vector, circ_DOCK1 overexpression vector, circ_DOCK1 overexpression vector + miR-NC, or miR-409-3p mimic before treatment of H_2_O_2_ or not. *P < 0.05.

To analyze whether miR-409-3p was required for circ_DOCK1 to regulate HBVSMC injury, HBVSMCs were transfected with vector, circ_DOCK1 overexpression vector, circ_DOCK1 overexpression vector + miR-NC, or miR-409-3p mimic prior to exposure to H_2_O_2_. After the transfection, miR-409-3p expression was markedly reduced by circ_DOCK1 overexpression, which was rescued *via* addition of miR-409-3p mimic (Figure 4A). Moreover, miR-409-3p upregulation abolished the influence of circ_DOCK1 on cell proliferation and PCNA expression in HBVSMCs under H_2_O_2_ (Figures 4B,C). Additionally, miR-409-3p overexpression reversed the influence of circ_DOCK1 on apoptosis and abundances of related proteins (Bcl-2, Bax, and cleaved PARP) in H_2_O_2_-treated HBVSMCs (Figures 4D,E). These results indicated that circ_DOCK1 modulated H_2_O_2_-induced HBVSMC damage by targeting miR-409-3p.

### MCL1 Is Targeted by miR-409-3p and Modulated *Via* circ_DOCK1/miR-409-3p Axis

**FIGURE 5:**
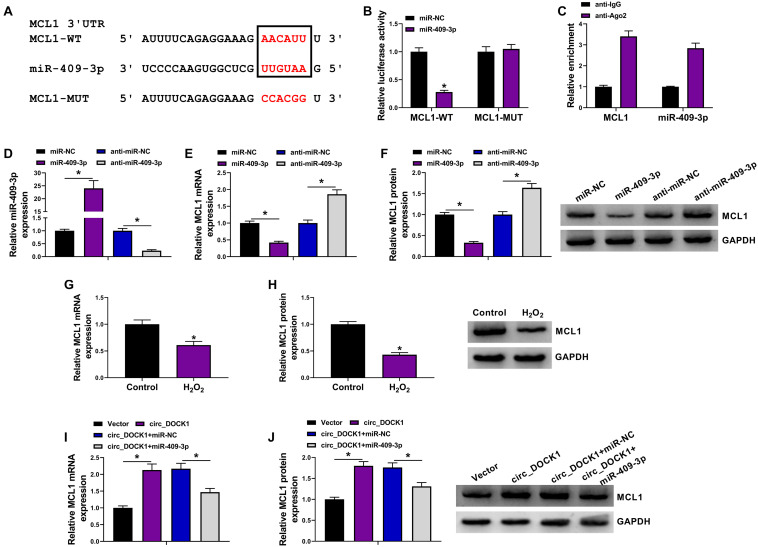
The target relationship of miR-409-3p and MCL1 in HBVSMCs. **(A)** The binding sequence of miR-409-3p and MCL1 was predicted using starBase. **(B)** Luciferase activity of MCL1-WT and MCL1-MUT vectors was detected in HBVSMCs with transfection of miR-NC or miR-409-3p mimic. **(C)** MCL1 and miR-409-3p enrichment levels were measured after Ago2 RIP. **(D–F)** MiR-409-3p and MCL1 levels were examined in HBVSMCs transfected with miR-NC, miR-409-3p mimic, anti-miR-NC, or anti-miR-409-3p. **(G,H)** MCL1 abundance was detected in HBVSMCs after stimulation of H_2_O_2_ or not. **(I,J)** MCL1 expression was measured in HBVSMCs transfected with vector, circ_DOCK1 overexpression vector, circ_DOCK1 overexpression vector + miR-NC, or miR-409-3p mimic. *P < 0.05.

To further explore the regulatory network, the molecular targets of miR-409-3p were analyzed *via* starBase. MCL1 was a potential target, and the target sites of miR-409-3p on MCL1 are exhibited in Figure 5A. To confirm this interaction, the MCL1-WT and MCL1-MUT vectors were constructed. MiR-409-3p mimic caused significant loss of luciferase activity of MCL1-WT, but it did not change the activity of MCL1-MUT (Figure 5B), and lots of MCL1 and miR-409-3p could be enriched in Ago2-based complex (Figure 5C). Furthermore, the effect of miR-409-3p on MCL1 expression was investigated in HBVSMCs transfected with miR-NC, miR-409-3p mimic, anti-miR-NC, or anti-miR-409-3p. The overexpression or knockdown efficacy of miR-409-3p mimic or anti-miR-409-3p is validated in Figure 5D. In addition, MCL1 expression was markedly decreased *via* miR-409-3p overexpression and increased by miR-409-3p knockdown (Figures 5E,F). Moreover, MCL1 abundance in HBVSMCs was evidently decreased by treatment of H_2_O_2_ (Figures 5G,H). Additionally, the influence of circ_DOCK1 on MCL1 expression was analyzed in HBVSMCs transfected with vector, circ_DOCK1 overexpression vector + miR-NC, or miR-409-3p mimic. Results showed circ_DOCK1 overexpression significantly upregulated MCL1 expression, which was decreased by miR-409-3p overexpression (Figures 5I,J). These results indicated that circ_DOCK1/miR-409-3p axis could target MCL1.

### MiR-409-3p Knockdown Mitigates H_2_O_2_-Induced HBVSMC Injury by Regulating MCL1

**FIGURE 6:**
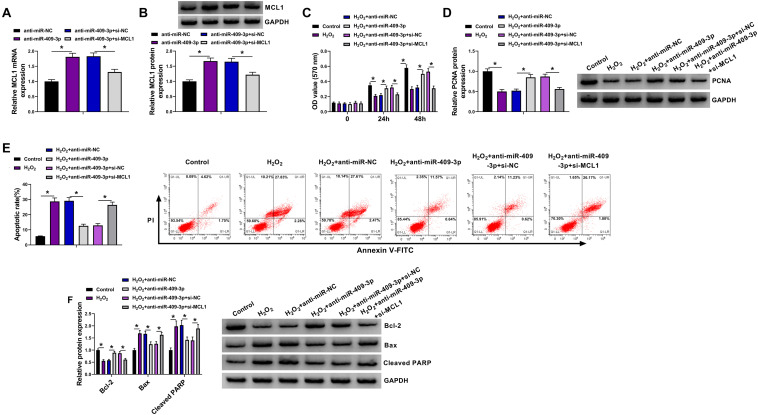
The effect of miR-409-3p and MCL1 knockdown on proliferation and apoptosis of HBVSMCs under H_2_O_2_. **(A,B)** MCL1 abundance was determined in HBVSMCs transfected with anti-miR-NC, anti-miR-409-3p, anti-miR-409-3p + si-NC, or si-MCL1. Cell proliferation **(C)**, PCNA expression **(D)**, apoptosis **(E)**, and expression of Bcl-2, Bax, and cleaved PARP **(F)** were measured in HBVSMCs with transfection of anti-miR-NC, anti-miR-409-3p, anti-miR-409-3p + si-NC, or si-MCL1 before treatment of H_2_O_2_ or not. *P < 0.05.

To study the function of miR-409-3p/MCL1 axis in HBVSMC injury, HBVSMCs were transfected with anti-miR-NC, anti-miR-409-3p, anti-miR-409-3p + si-NC, or si-MCL1 prior to exposure to H_2_O_2_. MCL1 abundance was obviously enhanced by miR-409-3p knockdown in HBVSMCs, which was reduced *via* addition of si-MCL1 (Figures 6A,B). In addition, miR-409-3p knockdown attenuated H_2_O_2_-mediated proliferation inhibition by rescuing cell proliferation and PCNA level, and this function was abrogated *via* interference of MCL1 using si-MCL1 (Figures 6C,D). Moreover, miR-409-3p downregulation weakened H_2_O_2_-induced apoptosis by decreasing apoptotic rate and expression of Bax and cleaved PARP and increasing Bcl-2 abundance, and these events were reversed by interference of MCL1 (Figures 6E,F). These findings suggested that miR-409-3p regulated H_2_O_2_-induced HBVSMC damage by targeting MCL1.

## Discussion

**FIGURE 7:**
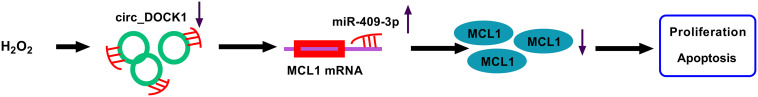
The schematic diagram of the circ_DOCK1/miR-409-3p/MCL1 axis in H_2_O_2_-induced HBVSMC injury. Circ_DOCK1 modulated miR-409-3p/MCL1 axis to regulate H_2_O_2_-induced apoptosis promotion and proliferation inhibition in HBVSMCs.

Intracranial aneurysm is local dilatation in cerebral arteries, and about 2–5% cases can develop to rupture (Xu et al., 2019). Smooth muscle cells are one key cell type-forming media in intracranial arteries and have significant roles in intracranial aneurysm formation and rupture (Starke et al., 2014). The apoptosis and impaired proliferation of brain vascular smooth muscle cells are related to intracranial aneurysm progression (Miyata et al., 2020; Wei et al., 2020). In our study, we established an *in vitro* model of intracranial aneurysm using H_2_O_2_-challenged HBVSMCs as previously reported (Zhao W. et al., 2018; Shi et al., 2019). We found that circ_DOCK1 could attenuate H_2_O_2_-induced apoptosis promotion and proliferation inhibition in HBVSMCs. Moreover, we provided a novel molecular explanation, the miR-409-3p/MCL1 axis, for the function of circ_DOCK1 (Figure 7). Such analysis was hampered at present by the lack of *in vivo* assays using the animal models of intracranial aneurysm.

Circular RNAs are relevant to vascular smooth muscle cell dysfunction and intracranial aneurysm development (Huang et al., 2019; Maguire and Xiao, 2020). Multiple evidences have reported that circ_DOCK1 could facilitate cell proliferation and constrain apoptosis in various cancers, like thyroid cancer, oral squamous cell carcinoma, bladder cancer, and colorectal cancer (Zhang et al., 2017; Wang et al., 2018; Liu P. et al., 2019; Cui and Xue, 2020). Moreover, circ_DOCK1 could increase proliferation of human umbilical artery smooth muscle cells (Wang et al., 2019). These all suggested the pro-proliferation and antiapoptotic functions of circ_DOCK1 in various cell lines. PCNA is a proliferation-related factor that regulates cell cycle process and DNA replication (Strzalka and Ziemienowicz, 2011). The antiapoptotic Bcl-2 and proapoptotic Bax are important players in intrinsic apoptosis (Cui and Placzek, 2018; Carpenter and Brady, 2021). PARP is a multifunction protein associated with DNA damage and cell apoptosis (Kumar et al., 2020). By combining the detection of these biomarkers, we found that circ_DOCK1 mitigated H_2_O_2_-driven proliferation inhibition and apoptosis promotion in HBVSMCs.

Next, we wanted to explore a regulatory network mediated by circ_DOCK1. Bekelis et al. (2016) identified 20 upregulated miRNAs in aneurysm tissues. After analyzing the interaction between them and circ_DOCK1 using CircInteractome, we found that only miR-409-3p had potential to bind to circ_DOCK1. Hence, we analyzed and confirmed that miR-409-3p was targeted by circ_DOCK1. Previous studies reported miR-409-3p could repress cell proliferation in papillary thyroid carcinoma, breast cancer, tongue squamous cell carcinoma, and osteosarcoma (Zhang et al., 2016; Chen and Dai, 2018; Zhao Z. et al., 2018; Wu et al., 2019). These reports all suggested the antiproliferation function of miR-409-3p in various cells. Similarly, our study validated the antiproliferation and proapoptotic roles of miR-409-3p in H_2_O_2_-treated HBVSMCs. Moreover, we further confirmed that circ_DOCK1 exhibited the protective function on H_2_O_2_-induced HBVSMC damage by inhibiting miR-409-3p.

We further explored the downstream targets of miR-409-3p, and found the anti-apoptotic MCL1 was targeted by miR-409-3p. Previous reports suggested that MCL1 could promote cell proliferation and inhibit apoptosis in pulmonary artery smooth muscle cells and rat thoracic aortic smooth muscle cells (Lee et al., 2015; Chen et al., 2018). Moreover, Zhao W. et al. (2018) showed MCL1 attenuated HBVSMC apoptosis by regulating the mitochondrial apoptotic pathway. Our results first identified MCL1 as a functional target of miR-409-3p. Furthermore, we first demonstrated that circ_DOCK1 could modulate MCL1 expression through miR-409-3p.

Previous work showed that H_2_O_2_ enhanced apoptosis of vascular smooth muscle cells depending on the regulation of miR-26a/PTEN/AKT/mTOR pathway (Peng et al., 2018). Moreover, Zhao W. et al. (2018) reported that H_2_O_2_ induced miR-29a expression in HBVSMCs and miR-29a knockdown abolished H_2_O_2_-dirven HBVSMC apoptosis, suggesting that H_2_O_2_ promoted HBVSMC apoptosis by upregulating miR-29a. Our data suggested that H_2_O_2_ drove HBVSMC apoptosis partially by regulating miR-409-3p/MCL1 axis *via* downregulating circ_DOCK1. With these findings, we envision that circ_DOCK1 may be a starting point for the development of circRNA-based therapies against intracranial aneurysm.

## Conclusion

In conclusion, circ_DOCK1 promoted cell proliferation and inhibited apoptosis in H_2_O_2_-treated HBVSMCs at least in part by regulating miR-409-3p/MCL1 axis. This study proposed the importance of circ_DOCK1/miR-409-3p/MCL1 axis in regulating HBVSMC dysfunction and provided a potential therapeutic target for intracranial aneurysm treatment.

## Data Availability Statement

The raw data supporting the conclusions of this article will be made available by the authors, without undue reservation, to any qualified researcher.

## Author Contributions

XD designed and performed the experiments and obtained the data. XW and LH performed the statistical analysis. ZZ and SJ wrote the sections of the manuscript. YT wrote the first draft of the manuscript. All authors contributed to manuscript revision, read, and approved the submitted version.

## Conflict of Interest

The authors declare that the research was conducted in the absence of any commercial or financial relationships that could be construed as a potential conflict of interest.

## Footnotes

1 https://circinteractome.nia.nih.gov/

2 http://starbase.sysu.edu.cn/
